# Ophthalmic involvement disparities in clinical characteristics of IgG4-related disease: a retrospective study of 573 patients

**DOI:** 10.1186/s12886-021-02210-z

**Published:** 2021-12-27

**Authors:** Linyang Gan, Xuan Luo, Yunyun Fei, Linyi Peng, Jiaxin Zhou, Jieqiong Li, Hui Lu, Zheng Liu, Panpan Zhang, Xiaowei Liu, Wen Zhang

**Affiliations:** 1 Department of Ophthalmology, Peking Union Medical College Hospital, Chinese Academy of Medical Sciences and Peking Union Medical College, Beijing, China; 2 Department of Rheumatology, Peking Union Medical College Hospital, Chinese Academy of Medical Science and Peking Union Medical College, National Clinical Research Center for Dermatologic and Immunologic Diseases (NCRC-DID), Beijing, China; 3 Department of Rheumatology and Immunology, The First Affiliated Hospital of Zhengzhou University, Zhengzhou, China

**Keywords:** IgG4-related disease, IgG4-related ophthalmic disease, Orbital manifestations, Orbit inflammation

## Abstract

**Purpose:**

To investigate the clinical manifestations of orbital involvement in a large cohort of Chinese patients with IgG4-related disease (IgG4-RD).

**Methods:**

A total of 573 patients with IgG4-related disease were included. We described and compared the demographic, clinical, laboratory and histopathologic findings from 314 patients with IgG4-related ophthalmic disease (IgG4-ROD) and 259 with extra-ophthalmic IgG4-RD.

**Results:**

Male predominance was found significant in extra-ophthalmic IgG4-RD only. Patients with IgG4-ROD showed younger age at diagnosis and longer duration from onset till diagnosis. In patients with extra-ophthalmic IgG4-RD, the most commonly involved extra-ophthalmic organ was pancreas; while in IgG4-ROD patients, salivary gland was most frequently affected. Multivariate analysis exhibited IgG4-ROD was associated with allergy history, higher serum IgG4/IgG ratio, multiple organs involvement and sialoadenitis. Orbital images were reviewed in 173 (55.1%) IgG4-ROD patients. Fifty-one (29.5%) patients had multiple lesions. Lacrimal gland involvement was detected in 151 (87.3%) patients, followed by extraocular muscles (40, 23.1%), other orbital soft tissue (40, 23.1%) and trigeminal nerve (8, 4.6%). Biopsy was performed from various organs in 390 cases. A dense lymphoplasmacytic infiltration and fibrosis were the main feature in orbital specimens. Storiform fibrosis and obliterative phlebitis were absent in lacrimal gland.

**Conclusions:**

Lacrimal gland involvement was the most common orbital manifestation of IgG4-ROD. Patients with IgG4-ROD showed different characteristic in demographic, clinical, laboratory findings compared to patients with extra-ophthalmic IgG4-RD. These features might indicate potential differences in the pathogenesis of these two subgroups of IgG4-RD.

**Supplementary Information:**

The online version contains supplementary material available at 10.1186/s12886-021-02210-z.

## Introduction

IgG4-related disease (IgG4-RD) is a recently recognized systemic fibro-inflammatory condition, histologically characterized by numerous lymphoplasmacytic infiltrations with a predominance of IgG4-positive plasma cells, storiform fibrosis and obliterative phlebitis in tumour-like lesions of multiple organs [1], usually accompanied with elevated serum IgG4 levels. Ocular tissues, including lacrimal gland, extraocular muscles, trigeminal nerve and orbital fat, are frequently affected by IgG4-RD [2, 3], forming the conception of IgG4-related ophthalmic disease (IgG4-ROD). It was initially defined as different entities, such as Mikulicz’s disease, idiopathic orbital inflammation or orbital benign lymphoid hyperplasia before the recognition of IgG4-RD [2].

In published cases series, the head and neck region is the second most common site for IgG4-RD after the pancreas [1, 4]. The frequency of ophthalmic involvement varies from 17 to 34% in IgG4-RD patients [5, 6]. Previous researches have suggested that IgG4-ROD patients may present different features compared with those with IgG4-RD without ocular lesions (extra-ophthalmic IgG4-RD). Lacrimal gland involvement is the most common ophthalmic manifestation of IgG4-RD and is frequently associated with extra-orbital diseases [5]. Wang et al. reported dacryoadenitis was an independent risk factor of relapse and raised the hazard ratio (HR) to 2.3 3[7]. Moreover, patients with the enlargements of extraocular muscles and trigeminal nerve exhibit a trend to have a higher risk for relapse as well [3].

Given its relatively recent recognition, most publications on this entity are case reports and small series. Larger case series are needed to better understand this rare disease and provide a higher level of evidence to improve patient care. Herein, based on data of 573 patients from our prospective cohort, we compared the clinical, laboratory, and pathological characteristics of 314 patients with IgG4-ROD and 259 with extra-ophthalmic IgG4-RD.

## Methods

A total of 573 IgG4-RD patients were enrolled in the cohort at Peking Union Medical College Hospital from 2011 to January 2020 (ClinicalTrials.gov ID: NCT01670695). All included patients fulfilled the 2019 ACR/EULAR classification criteria for IgG4-RD [8, 9]. Patients were excluded if they had infectious diseases, other rheumatic diseases, malignancies, or conditions that could mimic IgG4-RD. This study was approved by the Medical Ethics Committee of Peking Union Medical College Hospital. All patients provided written informed consent.

Patients’ age, sex, disease duration, allergy disease, and clinical manifestation were recorded. All patients underwent laboratory tests for complete blood count, erythrocyte sedimentation rate (ESR), hypersensitivity C-reactive protein (hs-CRP), serum immunoglobulin, IgG subclasses, total IgE levels, liver and renal function tests. Imaging examination, including computed tomography (CT), magnetic resonance imaging (MRI), positron emission tomography/computed tomography (PET-CT), or ultrasonography, were performed in some patients. In total, 378 patients (66.0%) underwent CT scan, 125 (21.8%) MRI, 99 (17.3%) PET-CT scan, and 171 (29.8%) ultrasound examination. Tissue biopsies were obtained (from 390 patients, 68.1%) and analyzed by pathologists.

IgG4-ROD was diagnosed based on ophthalmic manifestations and radiological findings, including enlargement of lacrimal gland, eyelid, trigeminal nerve, soft tissue and extra-ocular muscles. Extra-ophthalmic organ involvement was evaluated based on symptoms, signs, laboratory tests, imaging and histopathology. Disease activity was assessed by the IgG4-RD responder index (RI) [10].

Statistical analysis was performed using SPSS (version 20; IBM, Armonk, NY, USA). The normality of data distribution was confirmed using the Kolmogorov-Smirnov test. Continuous non-normally distributed data are presented as median (first quartile, third quartile) and assessed by non-parametric test. Categorical variables were analyzed by Fisher’s exact test or chi-square test. Stepwise multiple logistic regression analysis was performed to identify variables independently associated with the ophthalmic involvement. A *p*-value < 0.05 was considered statistically significant.

## Results

### Patients’ demographic characteristics

**Fig. 1:**
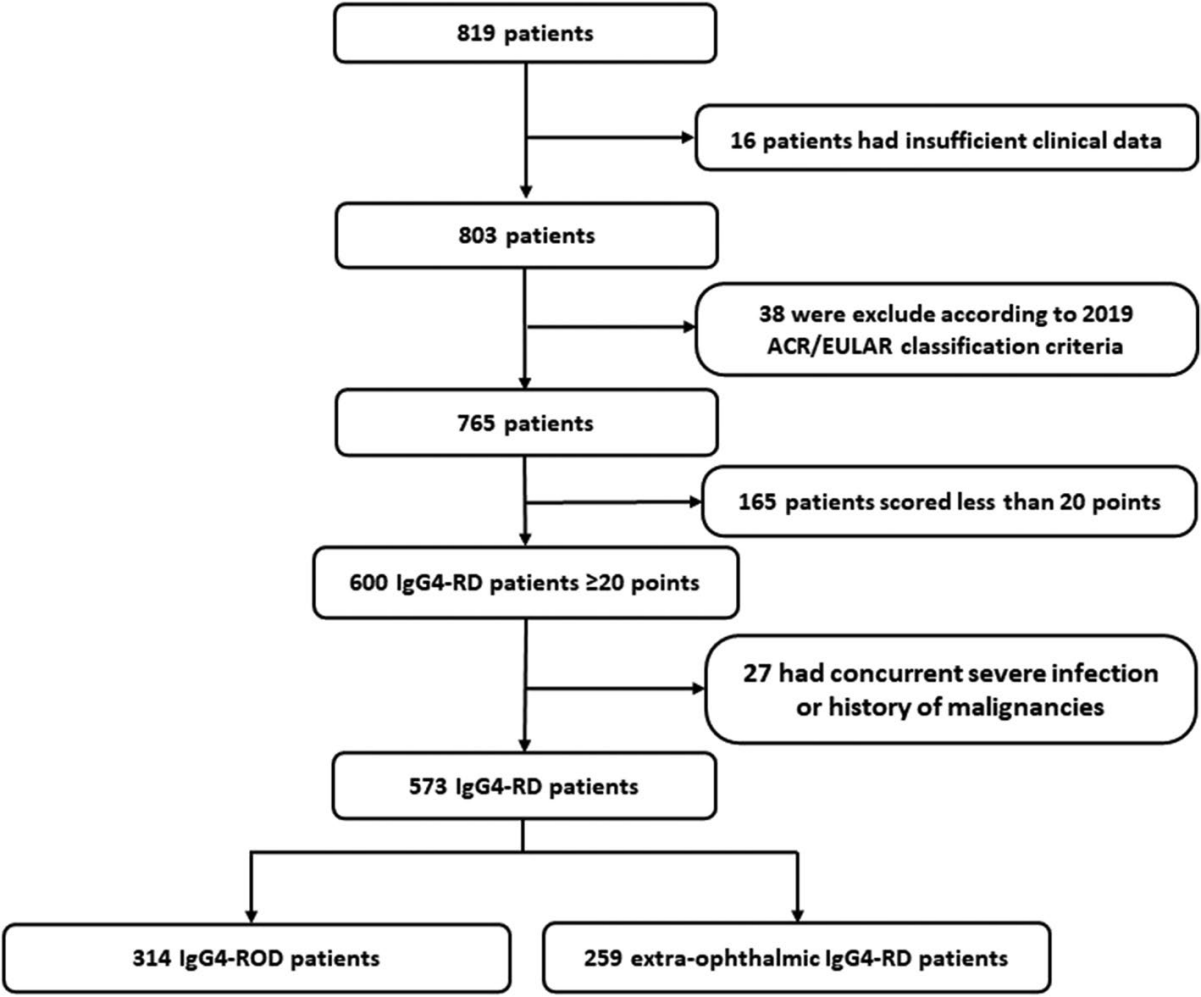
The flow diagram of included IgG4-RD patients

In total, 819 patients were screened and 246 of them did not enter the study due to the following reasons: 16 with insufficient clinical data for classification; 38 met the exclusion criteria of 2019 ACR/EULAR classification criteria; 165 whose total scores were less than 20 according to the 2019 ACR/EULAR classification criteria; another 27 with concurrent severe infection or the history of malignancies; the diagram was shown in Fig. 1. After screening, 573 eligible patients were enrolled in this study, including 314 with IgG4-ROD and 259 with extra-ophthalmic IgG4-RD. Patients’ demographic data and baseline clinical characteristics were shown in Table 1.

**Table 1:** Demographic features and baseline clinical characteristics of patients with IgG4-RD

Characteristics	All (***n*** = 573)	Extra-ophthalmic IgG4-RD (***n*** = 259)	IgG4-ROD (***n*** = 314)	***p***-value
**Male (%)**	62.30%	75.30%	51.60%	< 0.001
**Age, years (median, IQR)**	56 (47, 63)	59 (52,66)	53 (45,60)	< 0.001
**Time to diagnosis, months (median, IQR)**	12 (5, 36)	8 (3,24)	24 (12,52)	< 0.001
**Score of ACR/EULAR classification criteria (median, IQR)**	29 (25, 37)	27 (22,34)	31 (25,39)	< 0.001
**IgG4-RD RI (median, IQR)**	12 (6, 15)	12 (6,15)	12 (9,15)	< 0.001
**Symptoms at disease onset, n (%)**				
**Nausea and vomiting**	48 (8.4)	31 (12.1)	17 (5.4)	0.005
**Parotid gland swelling**	76 (13.3)	18 (7)	58 (18.1)	< 0.001
**Submandibular gland swelling**	264 (46.3)	83 (32.3)	181 (57.8)	< 0.001
**Lymphadenopathy**	138 (24.2)	56 (21.8)	82 (26.2)	0.222
**Abdominal pain**	95 (16.7)	72 (28)	23 (7.3)	< 0.001
**Jaundice**	92 (16.1)	71 (27.6)	21 (6.7)	< 0.001
**Nasal congestion**	98 (17.2)	23 (8.9)	75 (24)	< 0.001
**Cough**	57 (10)	29 (11.3)	28 (8.9)	0.354
**Low back pain**	30 (5.3)	16 (6.2)	14 (4.5)	0.351
**Fever**	28 (4.5)	19 (7.4)	9 (2.9)	0.013
**Allergy history, n (%)**	294 (51.3)	96 (37.1)	198 (63.1)	< 0.001
**Number of organs involved, n (%)**				< 0.001
**1–2**	165 (28.8)	109 (42.1)	56 (17.9)	
**3–4**	251 (43.9)	109 (42.1)	142 (45.4)	
**≥ 5**	156 (27.3)	41 (15.8)	115 (36.7)	
**Organ involvement, n (%)**				
**Salivary gland**	370 (64.6)	115 (44.4)	255 (81.2)	< 0.001
**Pancreas**	233 (40.7)	150 (57.9)	83 (26.4)	< 0.001
**Biliary system**	137 (23.9)	97 (37.5)	40 (12.7)	< 0.001
**Retroperitoneal fibrosis**	61 (10.6)	45 (17.4)	16 (5.1)	< 0.001
**Lung**	148 (25.8)	59 (22.8)	89 (28.3)	0.130
**Kidney**	73 (12.7)	31 (12)	42 (12.4)	0.615
**Lymph node**	261 (45.5)	115 (44.4)	146 (46.5)	0.616
**Sinusitis**	165 (28.8)	31 (12)	134 (42.7)	< 0.001
**Prostate**	51 (8.9)	33 (12.7)	18 (5.7)	0.003
**Thyroid gland**	15 (2.6)	8 (3.1)	7 (2.2)	0.521
**Gastrointestinal tract**	10 (1.7)	7 (2.7)	3 (1.0)	0.198
**Serum IgG4 elevation, n (%)**	524 (93.8)	233 (90.3)	301 (96.8)	0.001

***IQR*** **interquartile range**

The proportion of male sex in patients without ophthalmic involvement (75.3%) was higher than patients with IgG4-ROD (51.6%). Besides, patients with ophthalmic involvement were associated with younger age but longer time to diagnosis (median age at diagnosis: 53 vs. 59 years, *p* < 0.001; median duration from onset to diagnosis: 24 vs. 8 months, *p* < 0.001). Interestingly, there were 45 (7.9%,) patients (26 IgG4-ROD and 19 extra-ophthalmic IgG4-RD) whose onset symptoms occurred at age younger than 30 years (6–29 years); with ten patients younger than 18 years at disease onset.

### Clinical features

Clinical manifestations and organ involvement were evaluated systematically in all patients. Swelling of parotid gland (18.1% vs. 7%, *p* < 0.001), submandibular gland (57.8% vs. 32.3%, *p* < 0.001), and nasal congestion (24% vs. 8.9%, *p* < 0.001), were more common in patients with IgG4-ROD; while nausea and vomiting (12.1% vs. 5.4%, *P* = 0.005), abdominal pain (28% vs. 7.3%, *p* < 0.001), jaundice (27.6% vs 6.7%, *p* < 0.001), and fever (7.4% vs. 2.9%, *p* < 0.013) occurred more often in patients with extra-ophthalmic IgG4-RD. A higher proportion of IgG4-ROD patients reported a history of allergy (63.1% vs. 37.1%, *p* < 0.001). In addition, the baseline IgG4-RD RI of patients with IgG4-ROD was higher (*p* < 0.001), although the median values were both 12 in two groups.

More organs were affected in patients with IgG4-ROD (*p* < 0.001). In patients with IgG4-ROD, the most commonly involved organ second to ophthalmic tissue was the salivary gland (81.2%), followed by lymph nodes (46.5%) and paranasal sinuses (42.7%); while in patients with extra-ophthalmic IgG4-RD, the most commonly involved organs were pancreas (57.9%), salivary gland (44.4%) and lymph node (44.4%) (Table 1). Pediatric patients showed similar frequency of orbital involvement with adults (60.0% vs. 54.7%, *p* = 0.739). And other organ involvement in pediatric patients was also comparable with adults.

Orbital images were available in 173 (55.1%) IgG4-ROD patients (Fig. 3), with 135 (78.0%) cases showing bilateral involvement. Most patients (122, 70.5%) had single lesion, with 105 (86%) lacrimal gland, seven (5.7%) extraocular muscles, two (1.6%) trigeminal nerve and eight (6.6%) other soft tissue. Among 51 (29.5%) patients with multiple lesions, 46 (90.2%) cases exhibited lacrimal gland involvement, 33 (64.7%) extraocular muscles, six (11.8%) trigeminal nerve and 32 (62.7%) other soft tissue. Compared with multiple-lesions group, single-lesion group revealed a lower frequency of extraocular muscles (*p* < 0.001), trigeminal nerve (*p* = 0.009) and other soft tissue (*p* < 0.001) involvement. Higher frequencies of other anatomic locations affected were observed if lacrimal gland was spared (extraocular muscles, *p* < 0.001, χ2 test; trigeminal nerve, *p* = 0.01, Fisher’s exact test; other soft tissue, p < 0.001, χ2 test).

### Laboratory tests

**Fig. 2:**
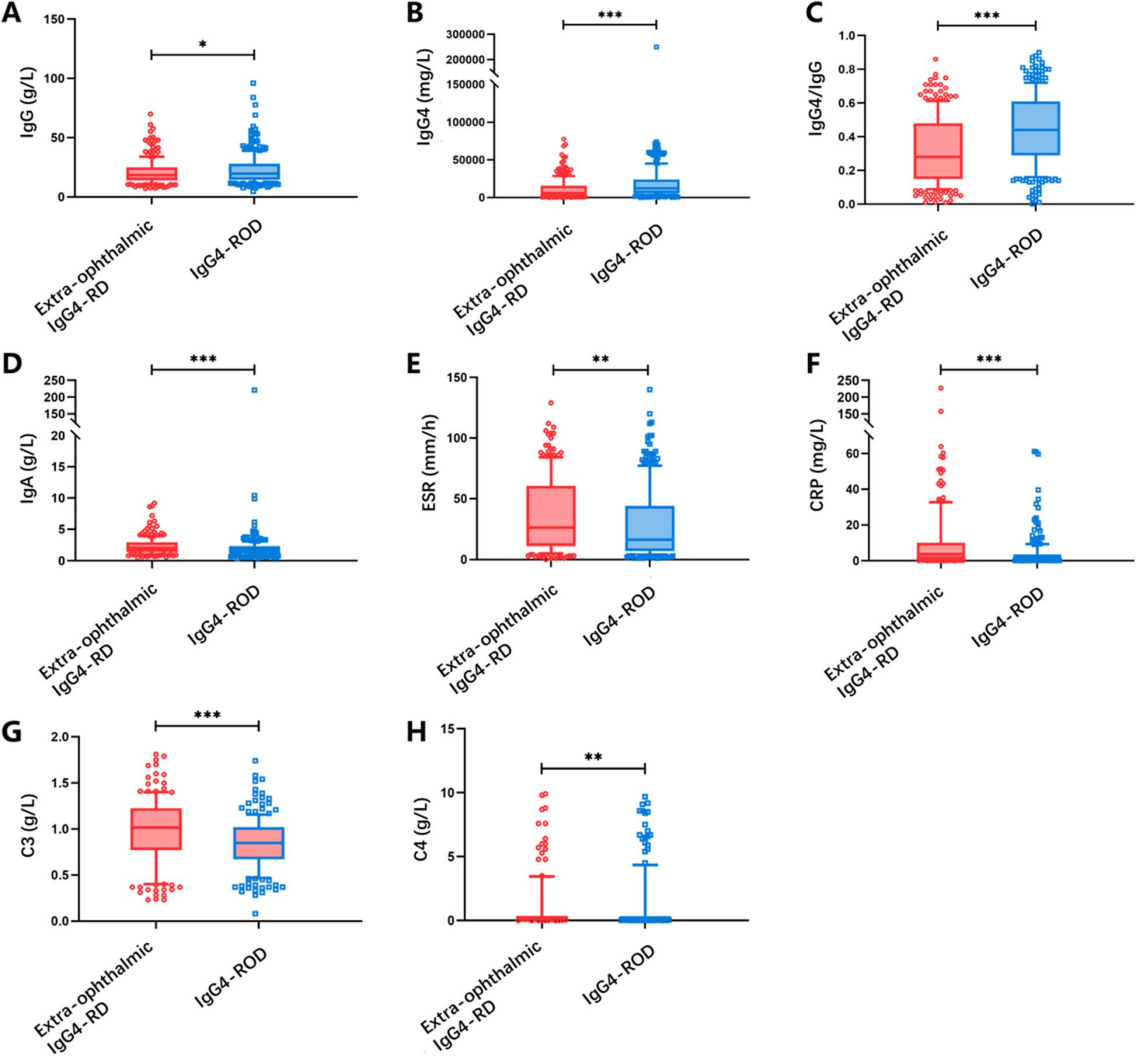
Laboratory findings of patients with IgG4-ROD and extra-ophthalmic IgG4-RD. Boxplots of baseline levels of (**A**) IgG, (**B**) IgG4, (**C**) IgG4/IgG ratio, (**D**) IGA, (**E**) ESR, (**F**) CRP, (**G**) C3 and (**H**) C4

Serum IgG4 levels elevation was found in 524 patients (93.8%). The proportion was higher in IgG4-ROD group (301, 96.8%) than extra-ophthalmic IgG4-RD group (233, 90.3%, *p* = 0.001). Laboratory findings of patients with IgG4-ROD and extra-ophthalmic IgG4-RD were compared as shown in Fig. 2. IgG4 level and IgG4/IgG ratio were higher in patients with IgG4-ROD (median IgG4 12.30 vs. 6.02 g/L, *p* < 0.001; median IgG4/IgG ratio 43.5% vs. 27.9%, *p* < 0.001, respectively). Patients with IgG4-ROD were found to have lower C3 level (*p* < 0.001), C4 level (*p* = 0.006), ESR (*p* = 0.001), CRP (*p* < 0.001) and IgA (*p* < 0.001) levels, but higher IgG levels (*p* = 0.047). Total IgE levels (*p* = 0.973) and eosinophils (*p* = 0.227) were comparable with patients without ophthalmic involvements (see supplementary figure).

### Association factors with ophthalmic involvement

In multivariate logistic analysis (Table 2), IgG4-ROD was found to be positively associated with allergy history (OR = 1.951, *p* = 0.016), higher ACR/EULAR Score (OR = 1.053, *p* = 0.004), higher IgG4/IgG ratio (OR = 12.892, *p* < 0.001), more organs involved (OR = 4.731, *p* < 0.001), and presence of sialoadenitis (OR = 2.286, *p* = 0.022). However, ophthalmic involvement was negatively affected by male sex (OR = 0.359, *p* = 0.001), autoimmune pancreatitis (OR = 0.102, *p* < 0.001), sclerosing cholangitis (OR = 0.047, *p* < 0.001), retroperitoneal fibrosis (OR = 0.019, *p* < 0.001), lung disease (OR = 0.130, p < 0.001), kidney disease (OR = 0.158, *p* < 0.001), and prostatitis (OR = 0.048, *p* < 0.001). Most laboratory findings, such as eosinophil, total IgE, C3, C4, ESR, CRP, showed no significant difference between the two groups.

**Table 2:** Multivariate logistic regression analysis of variables independently associated with IgG4-ROD

Variables	OR (95% CI)	***P***-value
**Sex (male)**	0.359 (0.197, 0.655)	0.001
**Allergy history**	1.951 (1.131,3.365)	0.016
**Score of ACR/EULAR classification criteria**	1.053 (1.017, 1.091)	0.004
**IgG4/IgG**	12.892 (3.101, 53.603)	< 0.001
**Number of organs involved**	4.731 (3.317, 6.748)	< 0.001
**Sialoadenitis**	2.286 (1.125,4.648)	0.022
**Autoimmune pancreatitis**	0.102 (0.048, 0.215)	< 0.001
**Sclerosing cholangitis**	0.047 (0.018, 0.123)	< 0.001
**Retroperitoneal fibrosis**	0.019 (0.005, 0.068)	< 0.001
**Lung disease**	0.130 (0.061, 0.280)	< 0.001
**kidney disease**	0.158 (0.062,0.401)	< 0.001
**Prostatitis**	0.048 (0.014, 0.160)	< 0.001

Age, sex, disease duration, IgG4 level, allergy history, number of organs involved, baseline IgG4-RD RI, Score of ACR/EULAR classification criteria and the presence of sialoadenitis, autoimmune pancreatitis, sclerosing cholangitis, sinusitis, retroperitoneal fibrosis, lung disease, kidney disease, and prostatitis were included for stepwise multiple logistic regression analysis

### Histopathologic findings of ophthalmic tissue

**Fig. 3:**
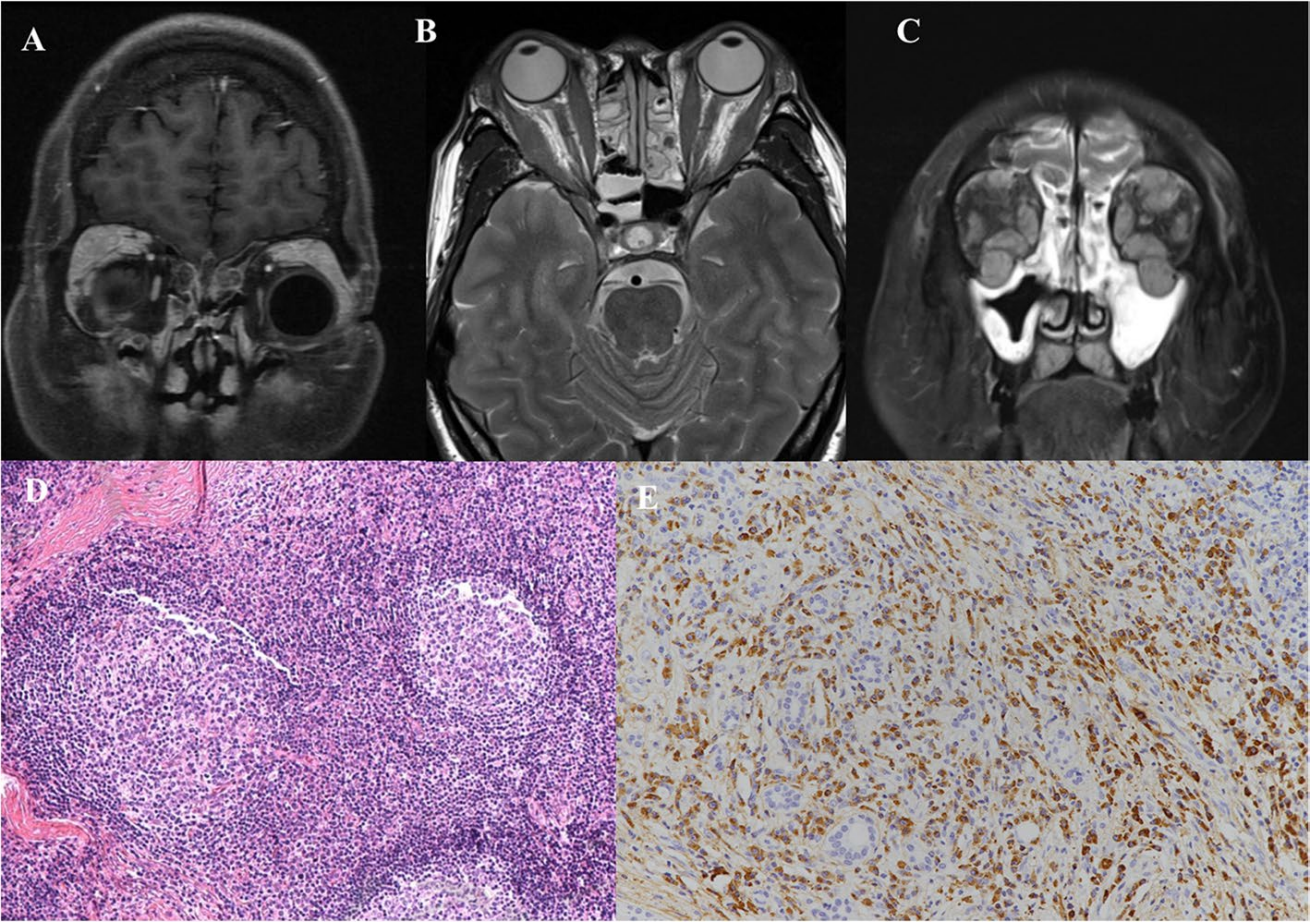
Radiological and pathological features of IgG4 related ophthalmic disease (IgG4-ROD). **A**-**C**: MRI scans showing lacrimal glands (**A**), extraocular muscles (**C** and **D**) and trigeminal nerves involvement (**C**). **D**-**E**: histopathological and immunohistochemical features of IgG4-ROD in a lacrimal gland biopsy: dense lymphocyte infiltration with fibrosis (hematoxylin and eosin) (**D**); immunohistochemistry for IgG4 (**E**)

Biopsy was performed from various organs in a total of 390 cases, mainly salivary gland. Fifty-three cases received ophthalmic biopsy. The specimens were consisted of 48 lacrimal gland samples, five specimens of orbital fat tissue, four specimens of skin tissue and two specimens of extraocular muscle. A dense lymphoplasmacytic infiltration and fibrosis were main features (Fig. 3). Storiform pattern fibrosis and obliterative phlebitis were not mentioned lacrimal gland samples. 60% specimens showed either an IgG4/IgG ratio > 40% or an IgG4+ plasma cells count > 10/HPF.

## Discussion

IgG4-RD patients with or without ophthalmic involvement showed different baseline demographic features. Patients with ophthalmic involvement were younger than patients with extra-ophthalmic IgG4-RD in this cohort. IgG4-RD primarily developed in a middle-aged population. Although pediatric cases are rare, IgG4-RD is now increasingly recognized to occur in children. Dozens of pediatric cases have been described [11]. Compared with adult patients, orbital manifestations are more frequently observed in children [12]. In this study, we included 10 pediatric cases, the youngest was 6 years old. However, we found no differences in organ involvement between children and adults. Larger pediatric cohorts are needed to confirm whether there are potential unique characteristics in certain age group.

Previous study has also revealed different clinical characteristics between female and male IgG4-RD patients [7]. IgG4-RD typically affects patients with a male to female ratio ranging from 1.6:1 to 4:1 [13, 14]. The sex ratio changes with patterns of organ involvement [15]. IgG4-related head and neck lesions seems to exhibit a more even sex distribution, while involvement of internal organs such as pancreas may be more frequent in male [16]. Here our data demonstrated that the male predominance tended to be more significant in patients with extra-ophthalmic IgG4-RD. But patients with IgG4-ROD exhibited a male-to-female ratio of 1:1, which was consistent with reports of other case series [17, 18].

In this cohort, the median time from onset to diagnosis was much longer in patients with IgG4-ROD (24 months). A longer delay in diagnosis was also reported in patients with major salivary gland involvement [19]. Patients with predominantly orbital involvement were more frequently misdiagnosed by ophthalmologists due to the lack of awareness of this rare disease. Furthermore, our multivariate analysis suggested that involvement of orbital tissue was negatively associated with autoimmune pancreatitis, sclerosing cholangitis, retroperitoneal fibrosis and other internal organ diseases, which might come with obvious symptoms that may remind patients to see a doctor. On the other hand, orbital manifestations are usually mild and not urgent, which may lead to a delay in seeking medical advice.

Organ involvement varies among different published case series. In studies of Thailand and India, orbits are commonly affected organs (34–58.6%) [6, 20]. While the leading affected organs reported in other populations include the pancreas in Singapore [21], Japan [22] and Italy [23],lymph nodes in Mexico [24] and France [5], and submandibular gland in China [19] and United States [14]. In this study, salivary gland was the most commonly affected organ (64.6%), followed by orbital tissues (54.8%). The submandibular, parotid and sublingual gland are often affected, with a frequency of 20–30% in Europe and 60–80% in Asia [8]. High frequency of lacrimal gland involvement is often associated with the involvement of salivary gland in the context of Mikulicz’s disease [15, 24].

With regard to ophthalmic lesions, lacrimal gland was the most frequently involved anatomic location of IgG4-ROD. Most studies reported that the frequency of lacrimal gland involvement varies form 66–83% [2, 5], which concurred with our results. Nevertheless, lesions in areas other than lacrimal gland were not uncommon. Extraocular muscles and trigeminal nerve branch enlargements were affected in 19–55% of patients with IgG4-ROD in previous studies [3, 5, 17] and 27.7% in this study. Some authors suggested that lesions in these areas might represent a progressive condition of IgG4-ROD and be refractory to systemic corticosteroid treatment [3]. Our data showed that involvement of areas other than lacrimal gland was associated with a high frequency of multiple types of lesions. This might indicate higher immune activity and a worse ophthalmic condition. Besides, lacrimal gland involvement seemed to be negatively related to other types of lesions. This suggested that instead of secondary infiltrations from affected lacrimal gland, lesions in these areas might be de novo lesions.

Serum IgG4 elevation was found in 93.8% IgG4-RD patients in this study. Published data also showed serum IgG4 elevation occurred in 55–97% of cases, especially in Asian patients [6, 17, 18]. The median IgG4 level in our patients with IgG4-ROD was 12.3 g/L, much higher than that of patients with extra-ophthalmic IgG4-RD of our cohort and patients with IgG4-ROD in literature [17, 18]. Although widely used as diagnosis criteria, elevation of serum IgG4 has poor diagnostic specificity. It can occur in a broad spectrum of systemic conditions, including infectious, neoplastic and autoimmune diseases [16]. Increased ratio of serum IgG4/IgG (> 10%) can increase diagnostic specificity [22], especially when IgG4 concentrations are only slightly raised. It is still controversial that whether serum IgG4 levels correlate with extra-ophthalmic involvement or higher relapse risk [17, 18]. However, our data suggested that higher IgG4/IgG ratio would be a better parameter indicating orbital involvement.

Other serological findings in patients with IgG4-RD are largely non-specific. Although patients with IgG4-ROD were found to have lower C3, C4, ESR, CRP and IgA levels than patients with extra-ophthalmic IgG4-RD, multivariate analysis detected no statistical significance. Hypocomplementemia at baseline may suggest kidney involvement [14]. An association between very low levels of C3 (< 50 mg/dl) and kidney involvement in IgG4-RD has been reported [15]. However, in our study, although C3 was lower in IgG4-RD patients with kidney involvement and IgG4-ROD, we did not detect any association between ophthalmic disease and kidney disease.

Histopathologic findings of IgG4-RD include three characteristic features: dense lymphocytic infiltration, storiform fibrosis and obliterative phlebitis [16]. In this study, dense lymphocytic infiltration was a general pathologic finding; while storiform fibrosis and obliterative phlebitis were described in only one skin specimen. Storiform fibrosis is a unique pattern associated with IgG4-RD [1]. However, it sometimes escapes from detection because the histological appearance is not always similar for all organs. Storiform fibrosis is absent within lacrimal gland, and the frequency of obliterative phlebitis is low in salivary gland and lacrimal gland [1, 16]. This can explain the low report rate of obliterative phlebitis and storiform fibrosis in our samples.

High number of IgG4+ plasma cells on immunohistochemistry is a disease hallmark [1]. Similar to histopathologic findings, the absolute number of IgG4 + plasma cells/HPF should be interpreted according to specific organs. For example, the cutoff value for salivary gland and lacrimal gland is recommended to be at least 100 cells/HPF; while that in the pancreas can be more than 50 cells/HPF and in lung > 20 cells/HPF [1, 16]. The ratio of IgG4+/IgG+ plasma cells must be at least 40% to support a histopathologic diagnosis of IgG4-RD. And higher numbers of IgG4+ plasma cells (typically 70% or higher) suggest a greater likelihood of IgG4-RD [1]. Thirty-two orbital specimens met the prevalent immunohistochemistry criteria in this study. However, only seven cases fulfilled the more restricted criteria (> 100/HPF). Higher cutoff value decreased diagnostic sensitivity dramatically in this study. Nevertheless, positive immunohistochemistry alone does not necessarily indicate IgG4-RD and should be correlated with other histologic and clinical features. Non-specific presence of IgG4+ cells in other inflammatory and neoplastic disorders was not uncommon [25, 26]. Finally, a IgG4/IgG ratio < 40% does not rule out the diagnosis of IgG4-RD, for the majority of samples from other organs in this study, including pancreas, lung, liver and paranasal sinus, IgG4/IgG ratios were less than 40%.

This study has limitations. Although it is based on a prospective cohort, the analysis itself is retrospective in nature. Some laboratory and histopathologic information was not available. And 44.9% of patients with IgG4-ROD needed additional orbital imaging studies to identify exact anatomic locations in detail. Finally, not all patients underwent thorough examinations of the whole body, some affected organs might remain undetected at initial evaluation.

In conclusion, we have described demographic, clinical, laboratory and histopathologic differences between patients with IgG4-ROD and extra-ophthalmic IgG4-RD based on a large cohort. Patients with IgG4-ROD were more even in gender and consisted of younger people with longer time from onset to diagnosis. Allergic history and multi-organ involvement were more common in the IgG4-ROD group. IgG4-ROD was positively associated with higher serum IgG4/IgG ratio and more frequent salivary gland involvement, but less internal organ involvement was observed. Lacrimal gland involvement was the most common manifestation of IgG4-ROD. But other anatomic areas were also frequently affected. These features might indicate potential differences in pathogenesis of these two subgroups.

## Supplementary Information

**Additional file 1.**

## Data Availability

Not applicable.
