# Supplementary material for: Ophthalmic involvement disparities in clinical characteristics of IgG4-related disease: a retrospective study of 573 patients
Source: BMC Ophthalmol. 2021 Dec 27;21:447. doi: 10.1186/s12886-021-02210-z (PMC8711185; doi:10.1186/s12886-021-02210-z)
Supplement: Supplementary file 1 — Additional file 1. [file 12886_2021_2210_MOESM1_ESM.docx]

**Clinical manifestations of orbital involvement in IgG4-related disease: a retrospective study of 573 patients**

Linyang Gan^1*^, Xuan Luo^2*^, Yunyun Fei^2^, Linyi Peng^2^, Jiaxin Zhou^2^, Jieqiong Li^2^, Hui Lu^2^, Zheng Liu^2^, Panpan Zhang^3^, Xiaowei Liu^1^, Wen Zhang^2^

* Linyang Gan and Xuan Luo contributed equally to this article and should be considered co-first authors.

1. Department of Ophthalmology, Peking Union Medical College Hospital, Chinese Academy of Medical Sciences & Peking Union Medical College, Beijing, China

2. Department of Rheumatology, Peking Union Medical College Hospital, Chinese Academy of Medical Science & Peking Union Medical College, National Clinical Research Center for Dermatologic and Immunologic Diseases (NCRC-DID), Beijing, China

3. Department of Rheumatology and Immunology, The First Affiliated Hospital of Zhengzhou University

**Correspondence:**

Xiaowei Liu, Department of Ophthalmology, Peking Union Medical College Hospital, Chinese Academy of Medical Science & Peking Union Medical College, Beijing, China; [waynepumch@163.com](mailto:waynepumch@163.com).

Wen Zhang, Department of Rheumatology, Peking Union Medical College Hospital, Chinese Academy of Medical Science & Peking Union Medical College, National Clinical Research Center for Dermatologic and Immunologic Diseases (NCRC-DID), Beijing China; [zhangwen91@sina.com](mailto:zhangwen91@sina.com)

**
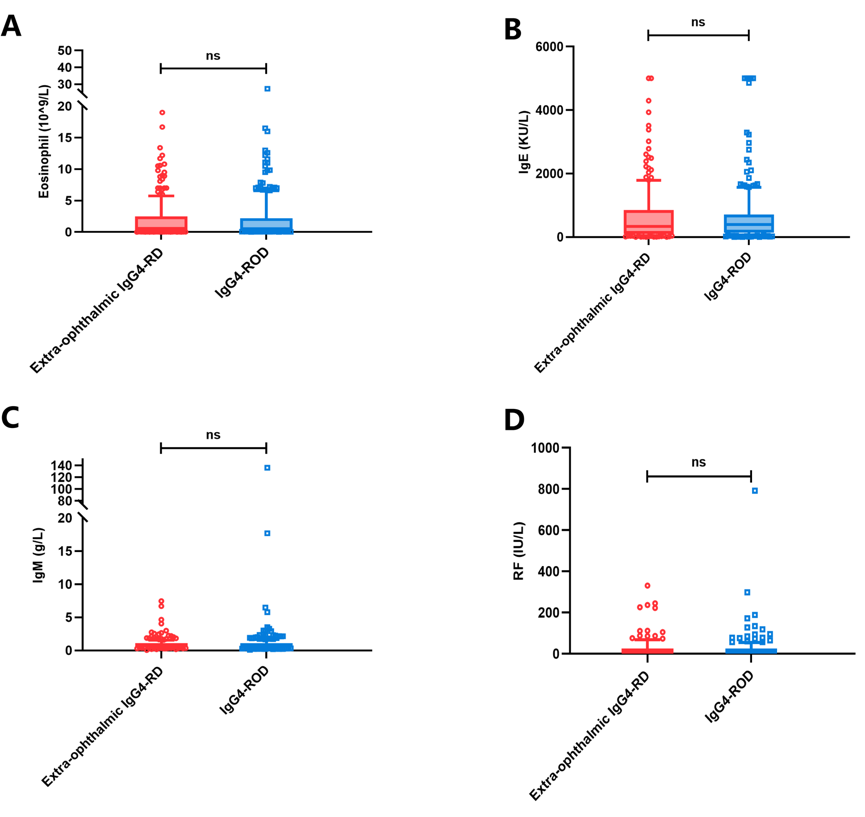
**

**Supplementary figure**

| **Table 1 Demographic features and baseline clinical characteristics of patients with IgG4-RD** | | | | |
| --- | --- | --- | --- | --- |
| **Characteristics** | **All (n=573)** | **Extra-ophthalmic IgG4-RD (n=259)** | **IgG4-ROD (n=314)** | ***p*-value** |
| **Male (%)** | 62.30% | 75.30% | 51.60% | <0.001 |
| **Age, years (median, IQR)** | 56 (47, 63) | 59(52,66) | 53(45,60) | <0.001 |
| **Time to diagnosis, months (median, IQR)** | 12 (5, 36) | 8(3,24) | 24(12,52) | <0.001 |
| **Score of ACR/EULAR classification criteria (median, IQR)** | 29 (25, 37) | 27(22,34) | 31(25,39) | <0.001 |
| **IgG4-RD RI (median, IQR)** | 12 (6, 15) | 12(6,15) | 12(9,15) | <0.001 |
| **Symptoms at disease onset, n (%)** |  |  |  |  |
| **Nausea and vomiting** | 48(8.4) | 31(12.1) | 17(5.4) | 0.005 |
| **Parotid gland swelling** | 76(13.3) | 18(7) | 58(18.1) | <0.001 |
| **Submandibular gland swelling** | 264(46.3) | 83(32.3) | 181(57.8) | <0.001 |
| **Lymphadenopathy** | 138(24.2) | 56(21.8) | 82(26.2) | 0.222 |
| **Abdominal pain** | 95(16.7) | 72(28) | 23(7.3) | <0.001 |
| **Jaundice** | 92(16.1) | 71(27.6) | 21(6.7) | <0.001 |
| **Nasal congestion** | 98(17.2) | 23(8.9) | 75(24) | <0.001 |
| **Cough** | 57(10) | 29(11.3) | 28(8.9) | 0.354 |
| **Low back pain** | 30(5.3) | 16(6.2) | 14(4.5) | 0.351 |
| **Fever** | 28(4.5) | 19(7.4) | 9(2.9) | 0.013 |
| **Allergy history, n (%)** | 294(51.3) | 96(37.1) | 198(63.1) | <0.001 |
| **Number of organs involved, n (%)** |  |  |  | <0.001 |
| **1–2** | 165(28.8) | 109(42.1) | 56(17.9) |  |
| **3–4** | 251(43.9) | 109(42.1) | 142(45.4) |  |
| **≥ 5** | 156(27.3) | 41(15.8) | 115(36.7) |  |
| **Organ involvement, n (%)** |  |  |  |  |
| **Salivary gland** | 370(64.6) | 115(44.4) | 255(81.2) | <0.001 |
| **Pancreas** | 233(40.7) | 150(57.9) | 83(26.4) | <0.001 |
| **Biliary system** | 137(23.9) | 97(37.5) | 40(12.7) | <0.001 |
| **Retroperitoneal fibrosis** | 61(10.6) | 45(17.4) | 16(5.1) | <0.001 |
| **Lung** | 148(25.8) | 59(22.8) | 89(28.3) | 0.130 |
| **Kidney** | 73(12.7) | 31(12) | 42(12.4) | 0.615 |
| **Lymph node** | 261(45.5) | 115(44.4) | 146(46.5) | 0.616 |
| **Sinusitis** | 165(28.8) | 31(12) | 134(42.7) | <0.001 |
| **Prostate** | 51(8.9) | 33(12.7) | 18(5.7) | 0.003 |
| **Thyroid gland** | 15(2.6) | 8(3.1) | 7(2.2) | 0.521 |
| **Gastrointestinal tract** | 10(1.7) | 7(2.7) | 3(1.0) | 0.198 |
| **Serum IgG4 elevation, n (%)** | 524(93.8) | 233(90.3) | 301(96.8) | 0.001 |
| **IQR: interquartile range** |  |  |  |  |

| **Table 2 Multivariate logistic regression analysis of variables independently associated with IgG4-ROD** | | |
| --- | --- | --- |
| **Variables** | **OR (95% CI)** | ***P*-value** |
| **Sex (male)** | 0.359 (0.197, 0.655) | 0.001 |
| **Allergy history** | 1.951 (1.131,3.365) | 0.016 |
| **Score of ACR/EULAR classification criteria** | 1.053 (1.017, 1.091) | 0.004 |
| **IgG4/IgG** | 12.892 (3.101, 53.603) | <0.001 |
| **Number of organs involved** | 4.731 (3.317, 6.748) | <0.001 |
| **Sialoadenitis** | 2.286 (1.125,4.648) | 0.022 |
| **Autoimmune pancreatitis** | 0.102 (0.048, 0.215) | <0.001 |
| **Sclerosing cholangitis** | 0.047 (0.018, 0.123) | <0.001 |
| **Retroperitoneal fibrosis** | 0.019 (0.005, 0.068) | <0.001 |
| **Lung disease** | 0.130 (0.061, 0.280) | <0.001 |
| **kidney disease** | 0.158 (0.062,0.401) | <0.001 |
| **Prostatitis** | 0.048 (0.014, 0.160) | <0.001 |
| Age, sex, disease duration, IgG4 level, allergy history, number of organs involved, baseline IgG4-RD RI, Score of ACR/EULAR classification criteria and the presence of sialoadenitis, autoimmune pancreatitis, sclerosing cholangitis, sinusitis, retroperitoneal fibrosis, lung disease, kidney disease, and prostatitis were included for stepwise multiple logistic regression analysis. | | |
